# Temporal Partitioning between Forest-Dwelling Small Rodents in a Mediterranean Deciduous Woodland

**DOI:** 10.3390/ani12030279

**Published:** 2022-01-23

**Authors:** Andrea Viviano, Manuel Scarfò, Emiliano Mori

**Affiliations:** 1 Consiglio Nazionale delle Ricerche, Istituto di Ricerca sugli Ecosistemi Terrestri, Via Madonna del Piano 10, 50019 Sesto Fiorentino, Italy; a.viviano@studenti.unipi.it; 2 Dipartimento di Scienze Agrarie, Alimentari e Agro-Ambientali, Produzioni Agroalimentari e Gestione degli Agroecosistemi, Università degli Studi di Pisa, Via del Borghetto 80, 56124 Pisa, Italy; 3 Dipartimento di Scienze della Vita e Biologia dei Sistemi, Università di Torino, Via Accademia Albertina 13, 10123 Torino, Italy; manuel.scarfo@gmail.com

**Keywords:** *Apodemus flavicollis*, *Clethrionomys glareolus*, camera-trapping, interspecific interactions, moon phases, temporal overlap

## Abstract

**Simple Summary:**

Camera-trapping has been widely used to assess activity rhythms and temporal overlap of different medium- and large-sized mammal species sharing the same habitats. Conversely, this method has been poorly applied to small mammals, which are often difficult to identify at the species level through photos. In our work, we assessed the temporal overlap between two coexisting small rodents in forest environments, *Apodemus flavicollis* and *Clethrionomys glareolus*. We collected 124 independent records of *A. flavicollis* and 67 records of *C. glareolus* over three years. The former was mostly nocturnal, with activity peaking after midnight, whereas the latter was mostly active at dawn and dusk. In other words, we recorded a limited temporal overlap, thus suggesting a potential for interspecific competition. Intraguild interference competition between *A.* flavicollis and *C. glareolus* may play a pivotal role, forcing *C. glareolus* to be more active in daylight hours, when the more strictly nocturnal *A. flavicollis* is present. Nocturnal activity of *C. glareolus* was limited and not influenced by moon phases, whereas *A. flavicollis* was mostly active in the darkest nights, avoiding bright moonlight nights.

**Abstract:**

Temporal partitioning is reported as one of the main strategies adopted by coexisting mammal species to limit interspecific competition and behavioural interference. In the last decades, camera-trapping surveys have provided valuable insights in assessing temporal niche and activity rhythms of medium and large-sized mammalian species. Conversely, this method has been poorly applied to small rodents. In this work we aimed at assessing temporal niche partitioning between two species of forest-dwelling small rodents—*Apodemus flavicollis* and *Clethrionomys glareolus*—by means of intensive camera-trapping. Camera traps were placed in areas where previous genetic analyses have confirmed the only presence of *A. flavicollis* amongst wood mice species, to prevent misinterpretation of records. We collected 124 independent records of *A. flavicollis* and 67 records of *C. glareolus* over three years. The former was mostly nocturnal, with activity peaking after midnight, whereas the latter was mostly active at dawn and dusk. Therefore, a limited temporal overlap was observed, confirming the potential for interspecific competition. Intraguild interference competition between *A. flavicollis* and *C. glareolus* may play a pivotal role forcing *C. glareolus* to be more active in daylight hours where, the more strictly nocturnal *A. flavicollis* is present. Nocturnal activity of *C. glareolus* was limited and not influenced by moon phases, whereas *A. flavicollis* was mostly active in the darkest nights, avoiding bright moonlight nights.

## 1. Introduction

Interspecific interactions have been widely reported to be one of the main factors shaping animal communities [1,2,3]. Interspecific competition may occur through interference, involving the prevention of use of a resource through direct physical interactions or resource destruction by one competitor, and, indirectly, though resource exploitation, involving the consumption of a resource and the reduction of its local availability [1,4,5,6]. Therefore, the overlap in the use of a certain resource between two or more species would trigger the potential for competition. Interference through direct physical aggressions mostly occur amongst mammalian carnivores [7,8], whereas herbivore species mostly compete with each other through resource exploitation [1]. Accordingly, amongst rodents, direct interspecific interference has been rarely observed, with most competition occurring through resource exploitation [9,10,11,12,13]. Together with diet, space and time are two major components of the ecological niche. Species sharing the same resource (i.e., potentially competing) may be adapted to coexist in an ecosystem by means of spatial, temporal or diet partitioning [9,14,15,16]. Assessing competition between small rodent species is difficult as species are often small nocturnal taxa, whose direct observation is challenging. Consistently, most studies on competition amongst rodents have been conducted on diurnal species [17,18,19,20], or on nocturnal species through long-term population dynamic studies based on capture-mark-recapture methods [21,22,23]. Activity rhythms of many small rodents have been determined through captures and time of trapping [24,25,26]. However, failures to trap animals does not necessarily imply lack of activity, which may in turn result in unreliable estimation of activity peaks [24,25]. Frequent trap-checks imply the repeated presence of an operator on the field, which may in turn force individuals to show unnatural patterns of activity [27]. Furthermore, when two competing species coexist, one might be easier to catch than another, thus distorting the estimation of interspecific overlaps of temporal activities [24,28].

In recent times, camera-trapping has been shown as a reliable method to estimate activity rhythms of wildlife, when a consistent number of records (i.e., over 30) is available for each species [29]. As for small rodents, camera-trapping has been mostly used to study activity patterns of easily identifiable species (e.g., diurnal species [30,31,32] or those showing unmistakable morphological features [33,34]). Conversely, this method has been poorly applied to small ground-dwelling rodents, possibly because of the difficulties in species identification from black/white videos [13,35,36]. Given the wide use of camera-trapping to assess activity rhythms of wild species including rodents [29], we think that this method, if kept active for 24 h and with cameras deployed at ground level to detect small species, would provide useful and reliable results.

In Mediterranean broad-leaved woodlands of Southern Europe, *Clethrionomys* voles and *Apodemus* wood mice are the predominant small rodent species, showing a wide spatial overlap [37,38,39,40,41]. In Italian forested areas, the yellow-necked wood mouse *Apodemus flavicollis* shows an almost complete distribution overlap with the bank vole *Clethrionomys glareolus* (formerly, *Myodes glareolus*: [42]). Competition amongst *A. flavicollis* and *C. glareolus* has been suggested, particularly after summer, when diet overlap increases [28,39,43]. In this context, *A. flavicollis* seems to be behaviourally dominant over *C. glareolus* [44,45]. Accordingly, anecdotal data also confirmed that most direct, aggressive behaviour occurs by *A. flavicollis* towards *C. glareolus* [28,46]. When food resources are abundant, competition between *A. flavicollis* and *C. glareolus* may be negligible [21,47]. This is consistent the fact that no injuries were observed in multiple capture events in Central Italy [28]. *Apodemus flavicollis* may use underground burrows created by *C. glareolus* [48], suggesting only a limited spatial partitioning. Microhabitat partitioning has been shown to occur between these species, which may limit competition occurrence, with *C. glareolus* selecting areas with a denser understory layer (or more open habitats, when food is scarce) with respect to *A. flavicollis* [21,43,49]. Temporal partitioning has also been suggested. Although both species are reported as predominantly nocturnal, *C. glareolus* may become more diurnal to limit encounters and to avoid aggressions by coexisting *A. flavicollis* [24,25,44]. Furthermore, *C. glareolus* seems to be mostly active in the bright moonlight nights, whereas *A. flavicollis* movements seem to increase in dark—or cloudy/rainy—nights [50]. However, detailed data on temporal overlap between these ground-dwelling rodents are not available, and only determined through trap-checks or in captive conditions, thus requiring further field studies. For this reason, the aims of our work were to determine the temporal overlap of activity patterns of *C. glareolus* and *A. flavicollis* by means of an intensive camera-trapping survey in a broad-leaved woodland of Central Italy. We predicted that (1) bank voles would show a more diurnal behaviour with respect to the yellow-necked mouse, and that (2) moonlight avoidance would occur in at least one of these species to limit encounters with the other.

## 2. Materials and Methods

### 2.1. Study Area

This work was carried out in the upper part of the Merse river valley (i.e., on the Metalliferous Hills of Central Italy). Our study area was located in the North-Eastern part of the province of Grosseto (Southern Tuscany), in a rural hilly area with a total area of about 1350 ha (43.087° N;10.986° E, 475–903 m a. s. l.), which includes a Site of Community Importance (Poggi di Prata: Tuscany Regional Law 56/2000). Most of the study area (about 67%) was covered by deciduous woodlands, mainly composed by *Quercus cerris* L., *Castanea sativa* Mill., *Ostrya carpinifolia* Scop., *Carpinus betulus* L., *Fraxinus ornus* L. and *Robinia pseudoacacia* L. Around these woodlands, there are belts of scrubwoods (*Juniperus* spp., *Rubus* spp., *Erica scoparia* L. and *Spartium junceum* L.: 1.71%) [51,52]. The climate shows sub-montane features. During our survey, average annual rainfall was 870–1000 mm and average annual temperature was 16 ± 3 °C (meteorological station of Campiano: www.idropisa.it. Accessed on 9 December 2021).

### 2.2. Camera Trap Survey

We collected field data between October 2018 and December 2021. Camera traps (Multipir 12 Scouting Camera) were placed at 12 stations (in a total area of 994 ha) (i.e., fixed georeferenced locations (trees or rocks), where each camera trap was tied with ropes and chains, separated from one another by at least 250 m). Given the small spatial extent of mice and vole movements (62–67 m: [53]), we assumed that no individual could have been present in more than one station, thus assuming independence between camera trap stations. All stations were located in coppiced woodlands (mostly *Quercus cerris* L. and *Castanea sativa* Mill.) showing similar microhabitats, temperature and altitude. Cameras were put on the closest path/track to predetermined random points selected in this habitat type through QGIS vers. 3.16.1 [54], within a regular grid. Cameras were placed at a height of ~0.1–0.3 m from the ground level, to improve the capture of small mammals, and oriented at 45°, pointing down, and 0.5 m from the side of the path.

Thigmotaxis is defined as the tendency of rodents to move close to walls or roots to limit predation risk [55]. To increase the capture success of small rodents, we preferred to orient our camera traps towards roots or rocks. They were kept active 24 h/day, to take 1 video (1 min) at each animal passage. Cameras were checked once every 10 days to download records and replace dead batteries. Each station was monitored through camera traps for at least 110 days/year.

### 2.3. Statistical Analyses

We defined “activity” as the cumulated period animals spend outside their shelter sites (e.g., trunk holes or underground burrows), regardless of their behaviour [29,56]. The close morphological resemblance makes the discrimination of *A. flavicollis* and *A. sylvaticus* impossible from camera-trap records [57,58]. Therefore, to limit bias and lack of reliability in our analyses, we conducted our camera-trapping survey in wooded areas where direct captures followed by genetics identification confirmed the presence of *A. flavicollis* only, amongst wood mouse species [52]. Conversely, the identification of *C. glareolus* from camera trap records was reliable as this is the only woodland vole present in our study area [51]. For all videos of *A. flavicollis* and *C. glareolus*, we recorded the date and the solar hour of capture (directly shown on the video). We pooled videos of both species in a single dataset, because data were not enough to allow seasonal analyses. We limited pseudoreplication bias by counting as one single “independent event” all videos of the same species taken by the same camera trap within the same 30 min period [29,33,59]. In other words, when more than one video of *A. flavicollis* or *C. glareolus* was recorded by the same camera trap in ≤30 min, we kept in our dataset only one record in the mid-time between the first and the last video. The Hermans–Rasson test (r test) was used to estimate whether *A. flavicollis* and *C. glareolus* exhibited a random activity pattern throughout the 24 h cycle [60]. It was computed through the package “CircMLE” [61], for the software R (version 3.6.1., R Foundation for Statistical Computing, Wien, Austria: www.cran.r-project.org. Accessed on 9 December 2021: [62]). We used the R package “overlap” [56] to assess activity rhythms and patterns of interspecific temporal overlap. We estimated the coefficient of overlap (Δ) between temporal activity patterns of *A. flavicollis* and *C. glareolus*. This coefficient ranges between 0 (no overlap) and 1 (total overlap: [56]). We used the Δ_4_ estimator, as the smallest sample of our pairwise comparison exceeded 75 records [56,63]. Afterwards, we computed the 95% confidence intervals of Δ_4_ (hereafter, 95% CI) by using 10,000 bootstrap replicates [64]. Overlap was considered as “intermediate” with Δ_4_ included between 0.50 and 0.75, “high” with Δ_4_ > 0.75, “very high” with Δ_4_ > 0.90 [65]. We also tested whether moon phases affected the activity of both ground-dwelling rodents by dividing surveyed nights into four moon-phase categories: (1) epact days = 0–3, 26–29; (2) epact days = 4–6, 21–25; (3) epact days = 7–9, 17–20; (4) epact days = 10–16. Then, we performed a chi-squared test on the numbers of videos recorded during each moon phase, to assess if they were uniform throughout the lunar cycle.

## 3. Results

**Figure 1:**
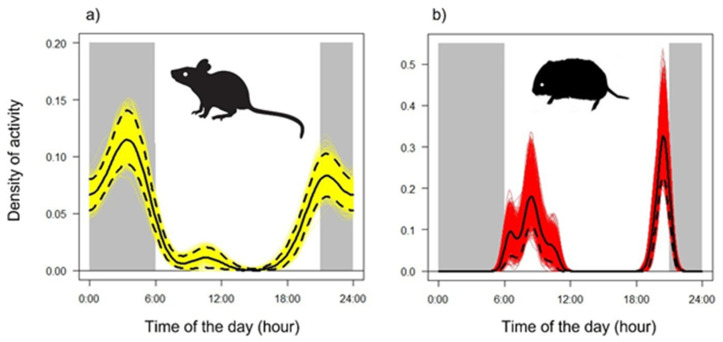
Patterns of activity rhythms of the forest-dwelling rodents: (**a**) *Apodemus flavicollis* and (**b**) *Clethrionomys glareolus*, expressed as Kernel density estimates of activity throughout the year. Black solid line—mean activity; dashed black lines—95% CIs; coloured (yellow and red) lines—bootstrap estimates. Dark bars show nocturnal hours.

We obtained a total of 124 independent records of *A. flavicollis* and 67 of *C. glareolus* in 4312 camera-nights (number of camera traps ∗ total nights of activity). Other 78 videos were discarded as it was not possible to identify the species with enough confidence. Activity patterns were significantly different from random according to the Hermans–Rasson test (*r* = 74.28–87.44, both *p* < 0.001) and activity peaked in the first part of the night (i.e., after sunset) for *A. flavicollis* (Figure 1a), at dusk and dawn for *C. glareolus* (Figure 1b).

**Figure 2:**
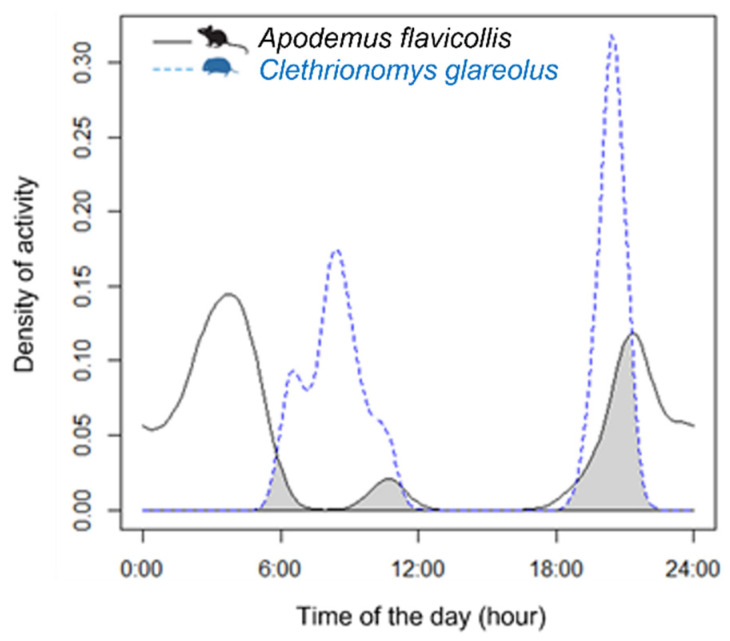
Annual overlap of activity rhythms between the *A. flavicollis* (solid black line) and *C. glareolus* (dotted blue line).

We observed a 22% overlap of activity rhythms between *A. flavicollis* and *C. glareolus* (Δ_4_ = 0.2266, 95% CIs = 0.2021–0.3536: Figure 2). Nocturnal activity of *C. glareolus* was limited and not influenced by moon-phases (c^2^ = 1.54, df = 3, *p* = 0.67), whereas *A. flavicollis* was mostly active in the darkest nights, avoiding bright moonlight (c^2^ = 53.67, df = 3, *p* << 0.01).

## 4. Discussion

Our results provided evidence of the effectiveness of camera-trapping to detect small mammals, although this method requires an intensive field effort (given that about 29% records were discarded because of species identification uncertainties) and a molecular verification of cryptic species (e.g., *Apodemus* wood mice). Absence of human presence and human smell may have allowed animals to perform their normal activities and to be indirectly monitored, thus providing reliable results on their normal activity rhythms [16,29,66,67].

The yellow-necked wood mouse was primarily nocturnal, with activity peaking in the second part of the night, in line with the previous literature based on individual captures [24,25] and with our prediction (1). Some diurnal activity has been observed only in the immediate surroundings of their burrows in the spring, when the nights are shorter and mice might be forced to range also in daylight hours to satisfy their needs (cf. [68] for the crested porcupine *Hystrix cristata*). The bank vole was instead mostly active in the morning and at dusk [24,69]. With our work, we provided reliable evidence of temporal partitioning between the yellow-necked mouse and the bank vole in a Mediterranean ecosystem without the bias induced by capture data only and by captive conditions (e.g., cafeteria experiment: [24,69]). However, this could represent the natural patterns for our focal species, and not the results of interspecific competition, which would require data from areas where *A. flavicollis* and *C. glareolus* are not syntopic. Although information on competition between *A. flavicollis* and *C. glareolus* are still partial and further field data are required [25,28,45,49], Gipps [70] reported that bank voles mediate their behaviour in presence of the yellow-necked wood mouse (e.g., by shifting their activity bouts from nocturnal to crepuscular hours). Furthermore, *C. glareolus* is reported to consume more green parts of plants (also including flowers and fruits), which may require a more diurnal activity to be visually detected, with respect to *A. flavicollis*, particularly in spring and summer [25,39]. If interspecific competition would be confirmed, time segregation would be much more evident when food is scarce (i.e., in late autumn and winter, when the interspecific diet overlap is the highest: [39]), or when population densities of at least one species are high [21]. Wróbel and Bogdziewicz [50] also suggested that weather conditions may represent a further way by which yellow-necked mice and bank voles segregate their temporal niche, with the former being mostly active in the darkest nights, including cloudy and rainy ones, and the latter mainly active in the brightest nights. Furthermore, together with most small rodents [26,33,71], *A. flavicollis* significantly avoided bright moonlight nights, possibly to limit its visibility to predators [72,73,74,75]. Moonlight avoidance has been previously suggested to occur for *Apodemus* species [14,50], but it has never been specifically tested before in *A. flavicollis*.

As to *C. glareolus*, nocturnal activity occurred irrespectively to moon phases. Accordingly, this species may avoid competitors and predators by increasing its ranging movement in daylight hours, i.e., when few predators (e.g., diurnal raptors and the pine marten *Martes martes*) are active (see [75] in the same study area). Its limited nocturnal activity occurs in early nights, thus being not affected by moon phase, confirming the results by [14]. Where nocturnal activity by *C. glareolus* is higher, this species is suggested to be more active in the bright moonlight nights [50], which is a common behaviour in mainly diurnal species exploiting night hours [76,77].

## 5. Conclusions

Our work provided evidence that, despite a high number of discarded records because of unreliable species identification, camera traps arrayed ad hoc (cf. Methods) may be used to estimate activity rhythms of small rodents. Temporal partitioning of activity rhythms amongst similar species has been suggested to occur to avoid intraguild interference competition [13,78]. This hypothesis has also been confirmed for ground-dwelling small rodents in wooded areas [44,69]. The bank vole and the yellow-necked wood mouse show differential temporal habits, which may represent a driver of coexistence based on the competitive dominance of the wood mouse. However, further studies where these species are not syntopic are needed to support this suggestion. Therefore, intraguild interference competition may play a pivotal role in this interspecific interaction, forcing bank voles to be more active in daylight hours (as well as in densely covered habitats) where yellow-necked mice are present. Other behavioural tactics (e.g., spatial partitioning at the microhabitat scale (with bank voles selecting dense understory sites)) have also been reported to limit potential interspecific interference, when *C. glareolus* and *A. flavicollis* search for the same food resource at the same time [21,43,49,69]. Future studies should be conducted to assess parasitic load of these species which may shape their host communities by apparent competition, affecting their spatiotemporal behaviour. Furthermore, the current presence of the Eurasian beaver *Castor fiber* on the Merse riverbanks [79], where this study has been carried out, may in the future influence population densities and behaviour of native small mammals, thus enhancing the importance of repeated field studies [80,81].

## Data Availability

Not Applicable.
